# Development and validation of machine learning for early mortality in systemic sclerosis

**DOI:** 10.1038/s41598-022-22161-9

**Published:** 2022-10-13

**Authors:** Chingching Foocharoen, Wilaiphorn Thinkhamrop, Nathaphop Chaichaya, Ajanee Mahakkanukrauh, Siraphop Suwannaroj, Bandit Thinkhamrop

**Affiliations:** 1 Department of Medicine, Faculty of Medicine, Khon Kaen University, Khon Kaen, 40002 Thailand; 2 Data Management and Statistical Analysis Center, Faculty of Public Health, Khon Kaen University, Khon Kaen, 40002 Thailand; 3 Department of Epidemiology and Biostatistics, Faculty of Public Health, Khon Kaen University, Khon Kaen, 40002 Thailand

**Keywords:** Rheumatology, Rheumatic diseases

## Abstract

Clinical predictors of mortality in systemic sclerosis (SSc) are diversely reported due to different healthcare conditions and populations. A simple predictive model for early mortality among patients with SSc is needed as a precise referral tool for general practitioners. We aimed to develop and validate a simple predictive model for predicting mortality among patients with SSc. Prognostic research with a historical cohort study design was conducted between January 1, 2013, and December 31, 2020, in adult SSc patients attending the Scleroderma Clinic at a university hospital in Thailand. The data were extracted from the Scleroderma Registry Database. Early mortality was defined as dying within 5 years after the onset of SSc. Deep learning algorithms with Adam optimizer and different machine learning algorithms (including Logistic Regression, Decision tree, AdaBoost, Random Forest, Gradient Boosting, XGBoost, and Autoencoder neural network) were used to classify SSc mortality. In addition, the model’s performance was evaluated using the area under the receiver operating characteristic curve (auROC) and its 95% confidence interval (CI) and values in the confusion matrix. The predictive model development included 528 SSc patients, 343 (65.0%) were females and 374 (70.8%) had dcSSc. Ninety-five died within 5 years after disease onset. The final 2 models with the highest predictive performance comprise the modified Rodnan skin score (mRSS) and the WHO-FC ≥ II for Model 1 and mRSS and WHO-FC ≥ III for Model 2. Model 1 provided the highest predictive performance, followed by Model 2. After internal validation, the accuracy and auROC were good. The specificity was high in Models 1 and 2 (84.8%, 89.8%, and 98.8% in model 1 vs. 84.8%, 85.6%, and 98.8% in model 2). This simplified machine learning model for predicting early mortality among patients with SSc could guide early referrals to specialists and help rheumatologists with close monitoring and management planning. External validation across multi-SSc clinics should be considered for further study.

## Introduction

Scleroderma or systemic sclerosis (SSc) is a rare connective tissue disease. Limited cutaneous systemic sclerosis (lcSSc) is characterized by skin tightness of the face, hands, feet, forearms, and legs, while diffuse cutaneous systemic sclerosis (dcSSc) is marked by skin tightness of the trunk and both extremities^1^. dcSSc is associated with internal organ fibrosis, and the prognosis is poor. dcSSc is more common among Thai SSc patients (70%) than lcSSc, which presents more commonly among Caucasians (17–37%)^2–4^.

Internal organ fibrosis among Thai sufferers of dcSSc presents more quickly, and the mortality rate is two times greater than that of lcSSc^5,6^. The most common cause of SSc-related vs. non-SSc-related death is cardiopulmonary involvement vs. pulmonary infection, respectively^5,7^. Overall survival among Thai SSc patients for either dcSSc or lcSSc^6^ is lower than that reported by Rubio-Rivas et al., who conducted a meta-analysis including 17 studies from Europe, the USA, North America, Australia, and Asia^5^. The lower survival among Thais is perhaps because (a) dcSSc with its poorer prognosis presents more frequently; (b) treatment options are limited; (c) drugs for pulmonary arterial hypertension (PAH) are prohibitively expensive; and/or (d) stem cell transplantation is unavailable.

The degree or severity of fibrosis is associated with poor prognosis in Thai SSc as well as other populations. Measures of severity include the extent of skin tightness (per the modified Rodnan skin score; mRSS) and internal organ involvements^5–8^. Mortality risk escalates the more numerous the involvements: 1 internal organ involvement had a 5 times greater risk than no involvements, while 2 internal organ involvements had a 10 times greater mortality risk^6^. These associations with fibrosis reflect the poor outcome of currently available treatments for halting the fibrotic process and/or mitigating its effects in patients with extensive disease.

According to the literature review, the various clinical predictors of mortality in SSc include older age at onset, dcSSc, positive for anti-Scl70 antibody, internal organ involvement (pulmonary fibrosis (PF), PAH, renal crisis, cardiac involvement), and high mRSS. The predictors vary with the inclusion/exclusion criteria and the experimental conditions in each of the cohorts/studies. Some predictors overlap, and some do not. We thus hypothesized that limiting the domain of our study to patients with other populations could create clinical prediction rules that could predict mortality among Thai SSc.

A previous study applied machine learning to predict pulmonary involvement in SSc from a small data set of 38 patients^9^. The authors found that the machine learning model could predict early pulmonary involvement, and so help identify the patients in early stages needing early treatment. Our study aimed to develop and validate a simplified predictive model from machine learning for mortality prediction among Thai SSc patients, making it a more precise referral tool for general practitioners, a guide for close monitoring, modification of the predictors of death, treatment planning, and minimizing mortality risk.

## Methods

### Patients

This study was prognostic research using a historical cohort study design. We included SSc patients over 18 years of age, diagnosed with SSc, attending the Scleroderma Clinic at Srinagarind Hospital, Khon Kaen University, Khon Kaen, Thailand, between January 1, 2013, and December 31, 2020. The data were extracted from the Scleroderma Registry Database. We excluded the patients lost to follow-up after the first visit and had a disease duration less than 5 years after onset. Data collection included the SSc subset, clinical characteristics of SSc, health status, date, and cause(s) of death (as appropriate). In addition, health status was requested from the Civil Registration Bureau if the patients were lost to follow-up.

**Figure 1:**
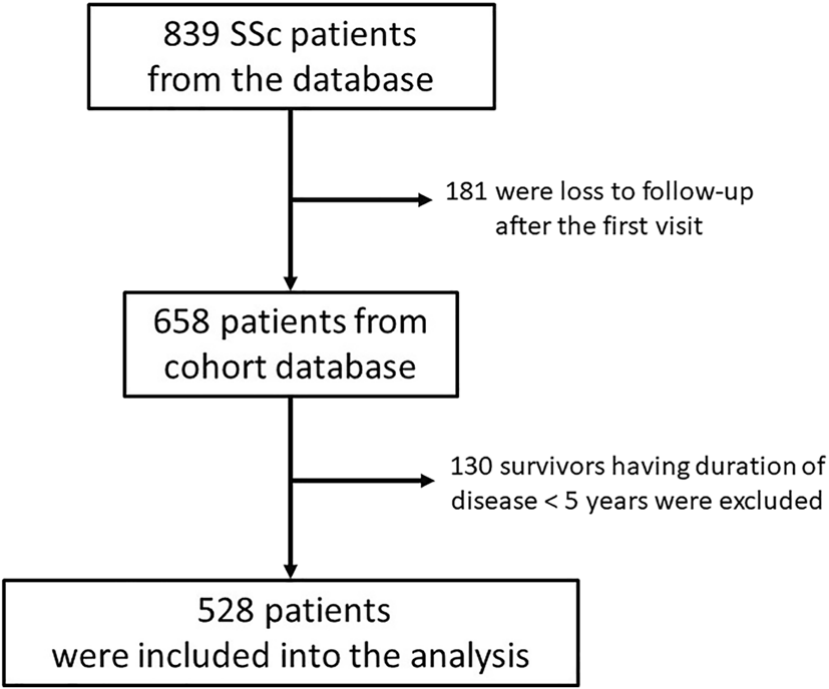
Flow of patients.

A total of 839 SSc patients from the database were evaluated—528 remained in the analysis after excluding the patients lost to follow-up after the first visit (181 cases) and having a disease duration less than 5 years after onset (130 cases) (Fig. 1).

### Operational definitions

All of the patients had a diagnosis of systemic sclerosis (SSc)—based on the American College of Rheumatology criteria and/or fulfilled the classification criteria of systemic sclerosis by ACR/EULAR 2013^10^. SSc was classified as the lcSSc or dcSSc subset as per LeRoy et al.^11^. The onset of the disease was the date of first non-Raynaud symptoms, while the end-date was the end of the study. Patients were censored if lost to follow-up or if they were still alive on the end-date. The time-to-event (death) was calculated by subtracting the end-date from the date of first SSc symptoms. Early mortality was defined when the patients died within 5 years after onset, with many deaths occurring in this period^6,12,13^. A digital ulcer was defined as a painful, denuded area with well-demarcated borders located on the volar aspect of the fingers^14^. Hand deformity was defined as the finger joints having flexion contractures resembling claw deformities^15^. The definition of PF was fulfilled when there were clinical signs and symptoms indicating PF (functional class II and above, crackles upon lung examination, cough, and/or abnormal chest radiography), and interstitial fibrosis was determined by high-resolution computed tomography (HRCT). Cardiac involvement was defined when the patients had congestive heart failure, poor left ventricular ejection fraction < 50%, or pericardial effusion. PAH was diagnosed when the mean pulmonary arterial pressure (mPAP) was > 20 mmHg at rest with a pulmonary artery wedge pressure of ≤ 15 mmHg with a pulmonary vascular resistance of ≥ 3 Wood units, as confirmed by right heart catheterization^16^. Esophageal involvement was defined when any esophageal symptoms of SSc were present (i.e., esophageal dysphagia, heartburn, or reflux symptoms). Stomach involvement was defined by early satiety or vomiting^17^. Intestinal involvement was determined if any of the following was present: diarrhea, bloating, malabsorption, constipation, and/or ileus or pseudo-intestinal obstruction. Myocardial involvement was defined when the left ventricular ejection fraction was ≤ 50%. A renal crisis was indicated when there was (a) a rapid, progressive rise in serum creatinine, (b) an abrupt onset of hypertension, and/or (c) microangiopathic hemolytic anemia. Weight loss was defined as the unintentional loss of > 5 percent of usual body weight over 6 to 12 months^18^. The definition of anemia was fulfilled if hemoglobin was < 12.0 g/dL in females and < 13.0 g/dL in males^19^.

### Predictive modeling

#### Prepare data

In the data set of 528 patients, four were removed due to missing data (neither mRSS nor WHO functional class were recorded), leaving 524 in the data set. Of the 23 SSc clinical features, only 4 (early mortality within 5 years, mRSS, World Health Organization (WHO) functional class ≥ II, and WHO functional class ≥ III) were selected for the analysis. Ninety-five of the 524 patients (18%) were non-survivors within 5 years after disease onset, while 82% survived to the end of the study. We tried to remedy the sample size imbalance by applying an autoencoder. Our aim was to develop a simple model in which only two features were used in each model. As a result, a few trainable parameters were included in the model training processes. The learning model only had a small number of predictive features, so imbalance might not have had much of an impact on accuracy. Hence, we also decided to perform data training with the original actual data with/without an autoencoder.

The data contained 23 clinical features for SSc. The 4 variables included in the analysis were dead within 5 years (dead5y), mRSS (mrss), WHO functional class ≥ II (fc2), and WHO functional class ≥ III (fc3). The fc2 and fc3 were transformed from the WHO functional class (fc) variables. Both fc2 and fc3 were set as binary data (0 or 1).

### Models with autoencoder

In order to avoid the limitation of dimensionality, due to having a small dataset with many features, any feature containing a variable group named “fc” that was used to transform to new ones (“fc2” and “fc3”) were excluded. As a result, the models that were being built only required three features (mRSS, fc2, and fc3). The above-mentioned imbalance data, however, needed to be handled properly. The autoencoder was then employed to address this issue. An Artificial Neural Network (ANN) is a type of autoencoder—a particular kind of neural network capable of learning a compressed representation of raw data^20,21^.

**Figure 2:**
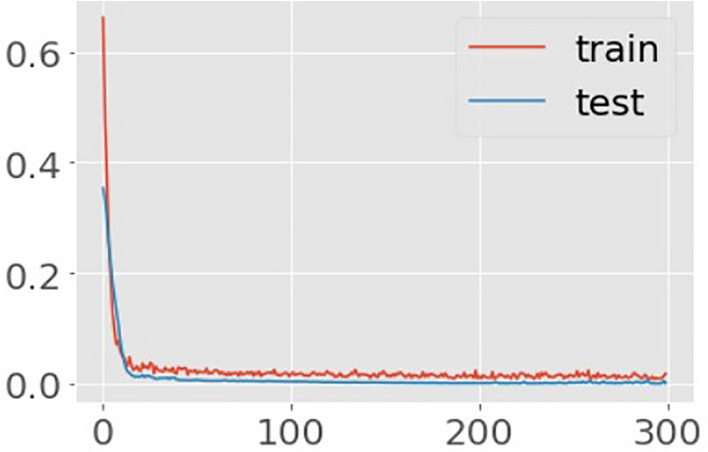
Learning curves of Autoencoder model (mRSS and WHO-FC ≥ II). Title = Autoencoder model loss. X-axis = Loss. Y-axis = number of epochs.

**Figure 3:**
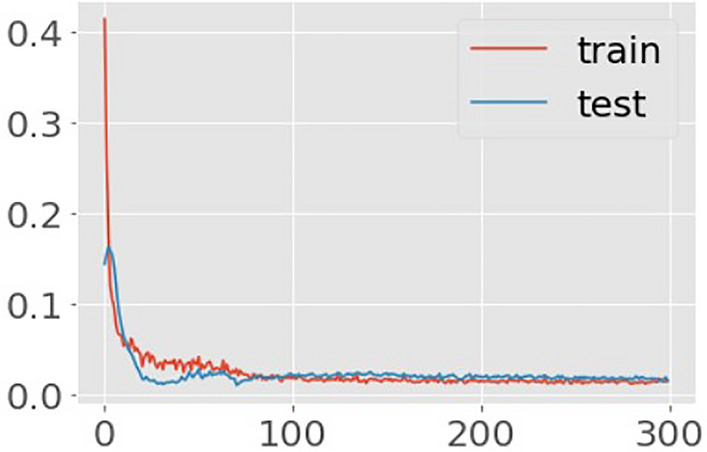
Learning curves of Autoencoder model (mRSS and WHO-FC ≥ III). Title = Autoencoder model loss. X-axis = Loss. Y-axis = number of epochs.

Encoder and decoder sub-models make up an autoencoder. The encoder compresses the input, and the decoder reconstructs the input from the encoder’s compressed form. Following training, the decoder model is abandoned, and the encoder model is saved. The satisfactorily reduced autoencoder error for the mRSS models with a WHO functional class II and III trained on the same target variable are shown in Figs. 2 and 3. These two plots of the learning curves show a good fit for reconstructing the inputs, which holds steady throughout training without overfitting either model (Figs. 2 and 3).

The reconstruction error in the autoencoder neural network model was determined by forecasting the test set to obtain the area under curve (AUC). First, the cut-point threshold was adjusted between 0.45 and 2, then the threshold of 0.55 was chosen to obtain a respectable confusion matrix with strong model performance. Finally, the encoder sub-models without the decoder sub-model were stored to be used in the other machine learning models in the study, including Logistic Regression, Decision Tree, AdaBoost, Random Forest, Gradient Boosting, and XGBoost.

The six machine learning models used in the dataset with autoencoder were similarly prepared. Using the proportions 0.7 (n = 366) and 0.3 (n = 158), the data were divided into training and test sets, respectively.

### Models without autoencoder

A real and original dataset was employed as an alternative approach to data preparation for the training models. Data containing the target variables—5-year dead and the three features (mRSS, WHO functional class II, and WHO functional III), which were the same features as the autoencoder model—were employed in the training phases. The Deep Artificial Neural Network and the six other machine learning models used this data. The data were divided by 0.2 for the test set (n = 105) and 0.8 for the training set (n = 419), which was also divided by 0.2 for validation of the deep learning model. The methods for developing simplified machine learning for mortality prediction for SSc patients are based on clinical, uncomplicated clinical assessments, and made available to general physicians. The WHO functional class II and mRSS have been reported as strong predictors of death in SSc from literatures^7,8^ and the variables need no special tests, are easily accessed, low-cost, and time-saving. Thus, both variables were employed to build models using machine learning and deep learning algorithms. The area under the ROC curve^22^ was constructed, and its 95% confidence interval (CI) estimated to demonstrate the model’s performance.

The deep learning models were validated using the validating set. The diagnostic performance of the model was estimated vis-à-vis sensitivity, specificity, positive predictive valuestogether with their 95% CIs. The Keras deep learning package with Tensorflow backend was used to implement the deep learning and other machine learning models in Python.

### Machine learning architecture

According to the mRSS continuous data (range score from 0 to 51), scaler transformation was performed with MinMaxScaler method for the entire dataset—for data with and without autoencoding. Other features—including fc2, fc3 and target—were binary variables, which were categorized as 0 or 1. When the WHO functional class was ≥ II, the fc2 was set to 1 and fc3 to 0, and when the WHO functional class was ≥ III, both fc2 and fc3 were set to 1. Binary cross entropy was assigned as a loss function, and the Adam optimizer was chosen for deep learning. Rectified Linear Unit (ReLU) was chosen as the activation function for transforming the summed weighted input node into the activation of three hidden nodes in a deep learning neural network, and sigmoid was chosen as the activation function for the output node. A deep learning model that has 400 epochs and 90 batch sizes was fitted. AUC was displayed following model evaluation, and model performance was given following prediction using the test set.

In addition, machine learning classifiers such as Logistic Regression, Decision tree, AdaBoost with decision trees (Adaptive Boosting), Random Forest, Gradient Boosting, and XGBoost were used to build different models.

The decision tree partitioned the data into two groups, then allocated classes into two groups (non-survivors/survivors) based on a majority vote. The best splitter of those two classes was determined using the entropy criterion. Additionally, the maximum depth of trees in AdaBoost, Decision Tree, Random Forest, and Gradient Boosting varied from 5 to 50. For these models with autoencoders, a maximum depth of 5 was chosen, and a maximum depth of 10 was chosen for models without autoencoders.

AdaBoost, Random Forest, Extreme Gradient Boosting, and XGBoost algorithms were developed sequentially, with each step designed to model the error of the preceding one by focusing on regularization to avoid overfitting, resulting in improved performance. For such models of both datasets, assigning the n estimator value to 20 was done before taking the maximum voting or averages of predictions, and a learning rate of 1.0.

### Ethics approval and consent to participate

The Human Research Ethics Committee of Khon Kaen University reviewed and approved the study as per the Helsinki Declaration and the Good Clinical Practice Guidelines (HE641219). Furthermore, all eligible patients signed informed consent before enrollment.

## Results

Females were more numerous than males (343 vs. 185 cases) for a female to male ratio of 1.9:1. The majority (374 cases; 70.8%) had the dcSSc subset. The median mRSS was 2 points (IQR 0–8). Around 59.4% of cases were in the WHO functional class ≥ II, and 14.9% were in WHO functional class ≥ III. PF was the most common organ involvement (45.9.6%), followed by esophageal involvement (41.7%) and intestinal involvement (15.6%). The overall clinical characteristics are presented in Table 1.

**Table 1:** Overall clinical characteristics.

Clinical characteristic	N = 528
Male sex (%)	185 (35.0)
Age at onset (years); mean ± SD	49.7 ± 11.9
Age (years); mean ± SD	59.4 ± 10.6
Diffuse cutaneous SSc subset (%)	374 (70.8)
mRSS (points); median (IQR)	2 (0–8)
WHO-FC ≥ II (%)	311 of 524 (59.4)
WHO-FC ≥ III (%)	78 of 524 (14.9)
Raynaud’s phenomenon (%)	253 (47.9)
Digital ulcer (%)	109 (20.6)
Salt and pepper skin appearance (%)	198 (37.5)
Calcinosis cutis (%)	27 (5.1)
Tendon friction rub (%)	67 (12.9)
Hand deformity (%)	203 (38.5)
Synovitis (%)	21 (4.0)
Pulmonary fibrosis (%)	241 of 525 (45.9)
Pulmonary arterial hypertension (%)	77 of 525 (14.7)
Cardiac involvement (%)	6 of 55 (10.9)
Renal crisis (%)	11 (2.1)
Esophageal involvement (%)	220 (41.7)
Intestinal involvement (%)	81 of 521 (15.6)
Weight loss (%)	76 (14.4)
Anemia (%)	294 (55.7)

*SD* standard deviation, *SSc* systemic sclerosis, *mRSS* modified Rodnan skin score, *IQR* interquartile range, *WHO* World Health Organization.

The clinical characteristics of alive and non-survivors are presented in Table 2. Factors more frequently found among the non-survivors were female sex, dcSSc subset, high WHO functional class, having tendon friction rub, renal crisis, esophageal involvement, and anemia. The non-survivors were older age at onset and had higher mRSS than survived cases. Two models gave the best performance based on the machine and deep learning. Model 1 included mRSS and WHO functional class ≥ II, while model 2 included mRSS and WHO functional class ≥ III. Most of the models with and without autoencoding provided accuracy and specificity greater than 80% and 90%, respectively. The AUC was highest from the deep learning model without autoencoding for both Model 1 and Model 2 with good precision and high specificity. Details of model performance from each method are presented in Table 3.

**Table 2:** Clinical characteristics of survived and non-survivors.

Clinical characteristics	Survive N = 433	Non-survive N = 95	Difference (95% CI)	p-value
Overall (%)	82.0	18.0		
Female sex; n (%)	300 69.3)	43 (45.3)	24.0 (13.1 to 34.9)	< 0.001
Age at onset (years); mean ± SD	48.1 (11.4)	57.2 (11.5)	− 9.1 (− 11.7 to − 6.1)	< 0.001
Age (years); mean ± SD	59.3 (10.5)	59.7 (11.3)	− 0.4 (− 2.8 to 1.9)	0.709
Diffuse cutaneous SSc subset; n (%)	298 (68.8)	76 (80.0)	− 11.2 (− 20.3 to − 2.0)	0.030
mRSS (points); median (IQR)	2 (0–4)	15 (4–29)	NA	< 0.001
WHO-FC ≥ II; n (%)	233 (54.2)	78 (83.0)	− 28.8 (− 37.7 to − 19.9)	< 0.001
WHO-FC ≥ III; n (%)	54 (12.6)	24 (25.5)	− 13.0 (− 22.3 to − 3.6)	0.001
Tendon friction rub; n (%)	47 (10.9)	20 (22.0)	− 11.0 (− 20.1 to − 2.0)	0.004
Pulmonary fibrosis; n (%)	202 (46.9)	39 (41.5)	5.4 (− 5.6 to 16.4)	0.343
Pulmonary arterial hypertension; n (%)	69 (16.0)	8 (8.5)	7.5 (1 to 14.1)	0.063
Cardiac involvement; n (%)	4 (8.7)	2 (22.2)	− 0.14 (− 0.42 to 0.15)	0.23
Renal crisis; n (%)	4 (0.9)	7 (7.37)	− 0.06 (− 0.12 to − 0.01)	< 0.001
Esophageal involvement; n (%)	160 (37.0)	60 (63.1)	− 0.26 (− 0.37 to − 0.15)	< 0.001
Intestinal involvement; n (%)	65 (15.2)	16 (17.6)	− 0.02 (− 0.11 to 0.06)	0.56
Anemia; n (%)	229 (52.8)	65 (68.4)	− 0.16 (− 0.26 to − 0.05)	0.01

*SD* standard deviation, *SSc* systemic sclerosis, *mRSS* modified Rodnan skin score, *IQR* interquartile range, *WHO-FC* World Health Organization functional class.

**Table 3:** Performance of the different algorithms.

Predictive model	Model performance					
	Accuracy (%)	AUC (%)	Precision (%)	Spec (%)	Recall (%)	F1-score (%)
**Model without autoencoder**						
**Deep learning models**						
Model 1 (mRSS and WHO FC ≥ II)	84.8	89.8	88.9	98.8	34.8	70.5
Model 2 (mRSS and WHO FC ≥ III)	84.8	85.6	88.9	98.8	34.8	70.5
**Other machine learning models**						
**Models for mRSS and WHO FC ≥ II**						
Decision Tree	81.9	77.0	45.5	93.1	27.8	34.5
AdaBoost	81.9	78.1	45.5	93.1	27.8	34.5
Random Forest	81.0	80.7	41.7	92.0	27.8	33.3
Gradient Boosting	81.9	78.7	45.5	93.1	27.8	34.5
XGBoost	81.9	81.0	45.5	93.1	27.8	34.5
Logistic regression	85.7	80.0	80.0	98.9	22.2	34.8
**Models for mRSS and WHO FC ≥ III**						
Decision Tree	83.8	66.0	53.8	93.1	38.9	45.2
AdaBoost	83.8	61.5	54.5	94.3	33.3	41.4
Random Forest	82.9	71.6	50.0	92.0	38.9	43.8
Gradient Boosting	83.8	66.3	53.8	93.1	38.8	45.2
XGBoost	82.9	74.5	50.0	93.1	33.3	40.0
Logistic regression	84.8	75.4	66.7	96.5	31.6	42.9
**Model with autoencoder**						
Model 1 (mRSS and WHO FC ≥ II)	82.3	73.0	62.5	92.7	44.1	70.4
Model 2 (mRSS and WHO FC ≥ III)	79.1	81.0	51.3	84.7	58.8	70.6
**Other machine learning models**						
**Models for mRSS and WHO FC ≥ II**						
Decision Tree	81.6	79.2	60.0	93.6	36.4	45.3
AdaBoost	81.6	79.4	60.0	93.6	36.4	45.3
Random Forest	82.3	80.3	61.9	93.6	39.4	48.1
Gradient Boosting	81.6	78.4	60.0	93.6	36.4	45.3
XGBoost	81.6	80.0	58.3	92.0	42.4	49.1
Logistic regression	82.9	80.8	80.0	98.4	24.2	37.2
Models for mRSS and WHO FC ≥ III	Results were not shown due to the modest variations between the models for mRSS and WHO FC II					

*AUC* Area under the receiver operating characteristics (ROC), *mRSS* modified Rodnan skin score, *WHO FC* World Health Organization functional class, *Spec.* Specificity, precision—positive predictive value, recall—sensitivity, *CV* cross-validation.

Two deep learning models were selected according to the best performance parameters for generating the predictive model of death. In order to simplify the model for practical use and avoid model overfitting, two clinical predictors were kept in the simplified model. Model 1 provided the same accuracy, positive predictive value, specificity, and specificity as Model 2 using the deep learning model without autoencoding (84.8%, 88.9%, 98.8%, and 34.8%, respectively). Meanwhile Model 1 gave a greater area under AUC than Model 2 (89.8 vs. 85.6). Table 4 presents the accuracy, area under ROC, positive predictive value, specificity, and sensitivity of Models 1 and 2.

**Table 4:** Generalizability of selected model(s) presented as accuracy, area under ROC, positive predictive value, specificity, and sensitivity.

Selected model	Accuracy	AUC (95%)	PPV (95%)	Specificity (95%)	Sensitivity (95%)
Model 1 mRSS and WHO FC ≥ II	84.8	89.8 (82.7–96.8)	88.9 (51.8–99.7)	98.8 (93.4–100.0)	34.8 (16.4–57.3)
Model 2 mRSS and WHO FC ≥ III	84.8	85.6 (74.9–96.2)	88.9 (51.8–99.7)	98.8 (93.4–100.0)	34.8 (16.4–57.3)

*95% CI* 95% confidence interval, *AUC* Area under the receiver operating characteristics (ROC), *mRSS* modified Rodnan skin score, *WHO FC* World Health Organization functional class.

**Figure 4:**
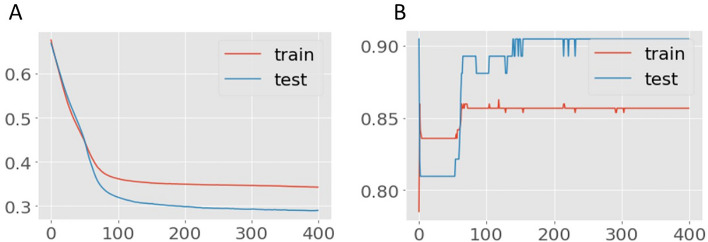
Learning curves of Model 1 (mRSS and WHO-FC ≥ II), (**a**) Training and validation loss, (**b**) the model accuracy in training and validation. Title = (**a**) Model loss (**b**) Model accuracy. X-axis = (**a**) Loss (**b**) Accuracy. Y-axis = (**a**) number of epochs (**b**) number of epochs.

**Figure 5:**
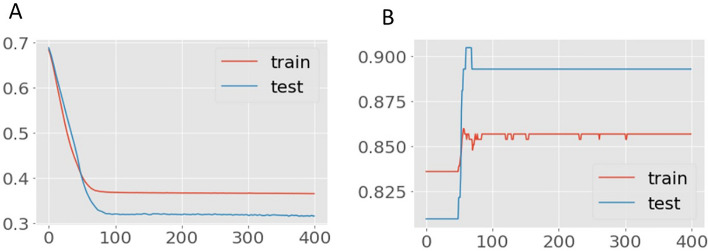
Learning curves of Model 2 (mRSS and WHO-FC ≥ III), (**a**) Training and validation loss, (**b**) the model accuracy in training and validation. Title = (**a**) Model loss (**b**) Model accuracy. X-axis = (**a**) Loss (**b**) Accuracy. Y-axis = (**a**) number of epochs (**b**) number of epochs.

**Figure 6:**
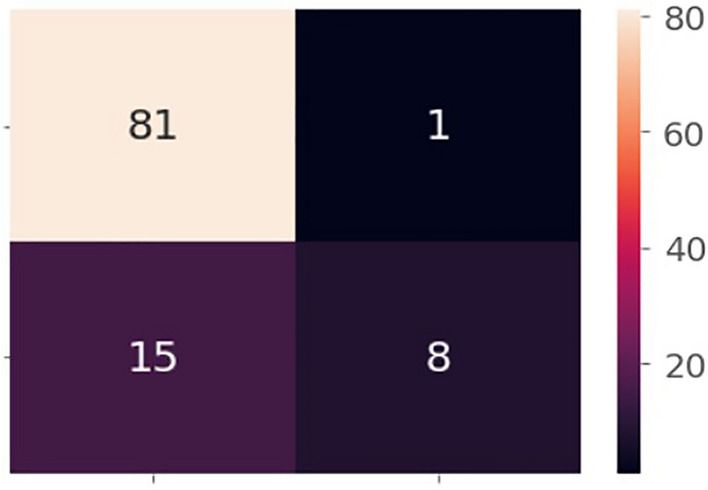
Confusion Matrix for both deep learning models without autoencoder. Title = Deep learning Confusion Matrix. X-axis (Left) = True Labels (upper row = Survivors, lower row = Non-survivors). X-axis (Right) = Number of cases. Y-axis = Predicted Labels (right column = Survivors, left column = Non-survivors).

Data sets divided for training, validation, and testing set in the deep learning models revealed that the network classifiers modified to predict survival or survived patients with SSc produced the best prediction compared to other machine models. The results of learning using the training procedures are presented in Figs. 4 and 5: the selected models represent learning curves that, after 50 epochs, had the best accuracy and lowest loss. Moreover, the confusion Matrix achieved for model mRSS + WHO functional class ≥ II and model mRSS + WHO functional class ≥ III had comparable performances (Fig. 6).

## Discussion

The mortality in SSc is higher than in the general population. The meta-analysis of Rubio-Rivas et al.^5^ showed that cumulative survival from diagnosis at 5 and 10 years was 74.9% and 62.5%, respectively. Currently, cardiopulmonary involvement is the leading cause of death in SSc^5–7^. Elderly age at the onset, male sex, extensive skin involvement, and internal organ involvement are well known predictors of death among SSc^5–8,23–26^. Various predictors are available for mortality in SSc as per differences in the cohort. A predictive model was previously developed using the European Scleroderma Trials and Research (EUSTAR) database^27^. The simplified score of the predictive model for the 3-year mortality in the study included the following factors: old age, male sex, dcSSc subset, renal crisis, dyspnea, digital ulcers, joint contracture, muscle weakness, high C-reactive protein, proteinuria, left ventricular ejection fraction (LVEF) < 50%, carbon monoxide diffusion capacity (DLCO) < 60% predicted, and forced vital capacity (FVC) < 70% predicted. The area under AUC was good (0.82)^27^; however, the model needed further investigation that might not be available at a primary center where the patient attends the first visit (LVEF, DLCO, and FVC). In addition, some clinical features are overlapped, which might have some collinearity by statistical analysis (i.e., dyspnea, FVC < 70% predicted, and LVEF < 50%). On the other hand, if the model were simplified, it could be used as a guide for the primary physician on managing the patients (i.e., close monitoring, early referral to a specialist).

Our study developed a simplified machine learning for mortality prediction for SSc patients based primarily on clinicals, which could be easily accessed, low cost, time-saving (unnecessary waiting for further investigation), and available for general physicians who are not experts in SSc. We proposed a model from machine learning to predict mortality in SSc. The model only included clinical features of patients and provided good test quality (i.e., area under ROC between 85.6 and 89.8). The model comprised two parameters (mRSS and WHO functional class), representing the severity of the disease. mRSS can represent the severity of skin involvement while a high WHO functional class indicates major organ involvement (viz., cardiopulmonary, renal, and/or musculoskeletal).

The deep learning model without autoencoding provided the highest accuracy, highest AUC, and highest precision for both Model 1 and Model 2 compared to the other machine learning methods. After applying an autoencoder to address the dataset imbalance, the accuracy and specificity were still high but the overall performance of the models remained inferior to the deep learning model without autoencoding. Model 1, which included mRSS + WHO functional class II gave higher accuracy and specificity but a lower AUC than Model 2, which included mRSS + WHO functional class III. Comparing the accuracy, positive predictive value, specificity, and sensitivity between Model 1 and 2, either model can be used to predict early mortality among SSc patients. In general, the WHO functional class III represents greater severity of cardiopulmonary involvement, implying that once a patient has a worsening WHO functional class, either Model 1 or 2 can be used for predicting mortality in SSc. However, Model 1 more correctly identifies those who will have a poor outcome. The survival prediction deep learning models in patients with SSc also followed the same path in both Models and generated the best prediction when compared to other machine models. The learning curves shown in Figs. 4 and 5 indicate that it takes approximately 50 epochs to reach the optimum accuracy and lowest loss. These models fared the best in terms of AUC, accuracy (positive predictive value), specificity, recall (sensitivity), and F1-score; although taking just somewhat longer to run than other models, they are sufficient and beneficial to deploy in the clinical context.

Generally, building a deep learning model is more costly in terms of time for designing the best architecture compared to other machine learning methods. The models chosen in this study can be scaled up quickly to deliver high accuracy with few errors. After 50 epochs, learning performance was already stable, long before the typical 400 epochs needed to fit a model. This indicates that model time spent learning is neither wasted nor a barrier.

The results from the deep learning model outperformed the autoencoder neural network model. This might be because autoencoder-based deep learning requires relatively more data to accurately classify study survivors and non-survivors than what is available. High performance from deep learning without an autoencoder may be attributable to the model's ability to train in a model with just two features that can make the best predictions without overfitting. Overfitting issues may arise if the model has more features. According to the epidemiology of SSc in Thailand, the most common SSc subset is dcSSc (70%)^6,28^, which explains why mRSS was included in the models in our study. Although not all primary physicians can conduct an mRSS assessment, severe skin tightness with scores of 2 or 3 are easily detected. Therefore, the mRSS might not be a significant problem when using the model.

Strengths of the study include: (1) the models comprised clinical features that represented the severity of skin involvement (mRSS) and internal organ involvement (WHO functional class), so the clinical features that predicted mortality from previous studies were included; (2) we included various machine learning models with and without autoencoding for predicting early mortality in SSc—the models provided various performances for predicting death in SSc patients in daily practice; (3) the scheduled follow-up visit and healthcare program were performed based on the attending physician, so the data represents the true nature of the cohort; and, (4) all patients in the database were from a single tertiary center that was included into the study; so, there was a low risk of selection bias. The limitations of our study were (1) some missing data, particularly renal crisis but the number of missing data of the others clinical features was low. According to our objectives, we would like to generate simplified machine learning from uncomplicated clinical features, so renal crisis was not included in our model even though there were missing data, and (2) our work mainly included the particular features of Thai patients with SSc among whom dcSSc is a common subset, so the model might not be generalizable. Notwithstanding, this prediction model (machine learning) can provide valuable data for Thai SSc patients, and perhaps, after follow-on validation, for other SSc patients in Southeast Asia. To generalize the models using external validation should be further studied. If the model is precise and accurate by external validation, we plan to test to a mobile application and/or website for general practitioners so that they can identify patients at risk of early mortality. General practitioners would then be able to direct patients to early referral, treatment planning, thereby minimizing mortality risk.

## Conclusion

Machine learning for predicting mortality in SSc using simple clinical features of SSc could guide attending physicians. Early awareness and referral of patients to a specialist would in turn help rheumatologists in monitoring and management planning. A model using predictors using high WHO functional class and mRSS appears to be optimal for predicting mortality risk among SSc patients. To generalize the model application, further study and external validation across multi-SSc clinics is needed.

## Supplementary Information

Supplementary Information.

## Data Availability

All data generated or analyzed during this study are included in this published article (supplementary file). In addition, the code for the models will be provided as an open source on GitHub after deployment of the model.
